# The Potential Use of Dynamics Changes of ctDNA and cfDNA in the Perioperative Period to Predict the Recurrence Risk in Early NSCLC

**DOI:** 10.3389/fonc.2021.671963

**Published:** 2021-07-16

**Authors:** Xiaolong Zhao, Fuqiang Dai, Longyong Mei, Depei Huang, Xudong Shen, Hushan Zhang, Xueke She, Zheng Ma

**Affiliations:** ^1^ The Department of Thoracic Surgery, Daping Hospital, Army Medical University, Chongqing, China; ^2^ The Medical Department, 3D Medicines Inc., Shanghai, China; ^3^ The Department of Thoracic Surgery, Chongqing General Hospital, University of Chinese Academy of Sciences, Chongqing, China

**Keywords:** non-small cell lung cancer, perioperative, ctDNA, cfDNA, predict

## Abstract

**Background:**

Postoperative circulation tumor DNA (ctDNA) is a promising method to predict the risk of recurrence. However, the amount of ctDNA in patients with early NSCLC is too small. Cell damages caused during the intraoperative period leads to a significant increase in cell free DNA (cfDNA). Whether cfDNA content is restored to the preoperative level within a short time after surgery may indicate the degree of surgical trauma. In this study, dynamic changes of cfDNA combined with ctDNA in the perioperative period of NSCLC were used to explore the possibility of them as a biomarker to indicate the risk of recurrence.

**Methods:**

NSCLC patients who planned to undergo radical resection were investigated. 10ml of peripheral blood was collected before, during and 7 days after surgery. DNA concentration was measured, and a 23-gene NGS panel was performed to detect gene mutations. All the patients would be followed-up for at least 18 months.

**Results:**

A total of 7 patients were sampled. The amount of cfDNA before surgery was 36.6 ± 14.7ng, and increased to 127.2 ± 52.2ng during surgery. 7 days after surgery, it dropped to 45.23 ± 9.41ng in 3 patients and rose to 173.7 ± 80.80ng in the remaining 4. Only 1 patient was ctDNA positive after surgery, with decreasing cfDNA, and he was the only one that relapsed and died within 18 months.

**Conclusion:**

The use of ctDNA to predict the risk of postoperative recurrence of NSCLC is a very valuable method, and it may be more reliable if combined with the dynamic changes of cfDNA. The amounts of cfDNA are raised by the operation, but will be polarized after surgery in 7 days. Postoperative NSCLC patients with positive ctDNA and reduced cfDNA have a higher risk of recurrence.

## Introduction

Lung cancer is one of the common malignant tumors, with the highest morbidity and mortality worldwide, 85% of which is non-small cell lung cancer (NSCLC) (1). Surgery is the most effective treatment, but 38-74% of patients will relapse within 5 years, and the risk of recurrence is associated with stage of the disease (2, 3).

cfDNA is a fragment of DNA released into the plasma after cell apoptosis by lysis, which carries genome-wide DNA information. ctDNA is a part of cfDNA, which can be derived from primary tumors and metastases, even circulating tumor cells (CTC). ctDNA also carries a full set of tumor genetic information and can effectively overcome the issues of the spatial heterogeneity of tumors. Therefore, liquid biopsy based on ctDNA has been widely used in the selection of treatment regiments of advanced NSCLC, including the identification of therapeutic drug targets, the detection of immunotherapy biomarkers, like blood tumor mutational burden (bTMB) and blood microsatellite instability (bMSI) (4, 5). ctDNA can also be used for early diagnosis and postoperative recurrence risk prediction in early NSCLC. However, due to the limited sensitivity of detection methods, its application scope is highly constrained. The ctDNA abundance of early NSCLC patients, especially for postoperative patients, is even lower than the detection limit of the conventional NGS detection method, which greatly reduces the detection sensitivity and causes false-negative results. Therefore, taking status of negative (undetected) or positive (detected) ctDNA after surgery as the basis for evaluation may underestimate the risk of recurrence. The release of cfDNA is associated with tissue trauma and stress reaction, both of them are associated with postoperative recurrence. Evidences showed that the level of cfDNA in tumor patients was higher compared with healthy people, and it significantly increased during tumor progression of non-surgical patients (6, 7). Therefore, the use of dynamic change of cfDNA combined with ctDNA as a biomarker might provide us a new approach to predict the risk of early NSCLC postoperative recurrence. In this study, the dynamic changes of cfDNA and ctDNA during the perioperative period of NSCLC were investigated to explore the possibility of them as a biomarker to indicate the risk of postoperative recurrence.

## Patients and Methods

### Patients

7 patients with NSCLC who were admitted to our hospital from May 2019 to August 2019 for radical surgery were investigated, including 3 males and 4 females, aged from 51 to 75 years old, with an average age of 65.00 ± 11.62 years old. Clinic TNM stage: 1 case of stage IA2, 3 case of stage IB, 1 case of stage IIA, 2 case of stage IIB (**Table 1**).

**Table 1:** Basic information of participants.

Patient No.	Gender	Age	ECOG	Lesion location	Pathological diagnosis	cTNM	pTNM	Adjuvant therapy
P01	F	75	0	Right upper apical lung	Invasive adenocarcinoma	cT2aN0M0, IB	pT1cN2M0, IIIA	pemetrexed + nedaplatin
P02	M	75	0	Posterior basal segment of the right lower lung	Squamous cell carcinomas	cT2bN0M0, IIA	pT2bN0M0, IIA	pemetrexed + nedaplatin
P03	F	51	0	Upper right lung	Invasive adenocarcinoma	cT3N0M0, IIB	pT3N2M0, IIIB	pemetrexed + nedaplatin
P04	M	74	0	Upper left lung	Squamous cell carcinomas	cT1bN0M0, IA2	pT1cN0M0, IA3	NA
P05	M	55	0	Upper right lung	Squamous cell carcinomas	cT3N0M0, IIB	pT3N0M0, IIB	pemetrexed + nedaplatin
P06	F	73	0	Upper right lung	Invasive adenocarcinoma	cT2aN0M0, IB	pT2aN0M0, IB	NA
P07	M	52	0	Lower right lung	Adenocarcinoma	cT2aN0M0, IB	pT2aN2M0, IIIA	pemetrexed + nedaplatin

### Methods

This study was approved by the ethics committee of Daping Hospital affiliated to Army Medical University. Each patient signed an informed consent before enrollment. 10ml peripheral blood was collected before the operation (within 2 hours before the operation, preoperative), immediately after the operation (intraoperative) and after the operation (7-10 days after the operation, postoperative), and surgically excised tissues were taken as control. DNA concentration was quantified by the Qubit dsDNA HS Assay Kit (Thermo Fisher Scientific). Illumina NextSeq-500 sequencing platform from 3D Medicines (Shanghai, China) was used to detect abundance of ctDNA and tissue genes. The 23-gene NGS panel including: *ALK, BCL2L11, BRAF, CDKN2A, DPYD, EGFR, ERBB2, FGFR2, KRAS, MET, NRAS, NRG1, NTRK1, NTRK2, NTRK3, PIK3CA, PTEN, RB1, RET, ROS1, TP53, TPMT, UGT1A1.*

## Results

### Surgical Results

All 7 patients had successfully undergone operations and completed blood sampling, the operation status is shown in **Table 2**. All patients were evaluated by pathologic TNM staging after surgery, and the results showed one case each of stage IA3, stage IB, stage IIA, stage IIB, stage IIIB, and two cases of stage IIIA (**Table 1**). All the patients with a stage II and stage III disease received 4 cycles of adjuvant chemotherapy (pemetrexed + nedaplatin).

**Table 2:** Surgical methods and results.

Patient No.	Resection margin	Operation method	Number of lesions	Tumor differentiation	Tumor size (cm)
P01	R0	Right upper lobectomy and lymph node dissection by single - port thoracoscopy	1	Unknown	3.0×3.0
P02	R0	Right lower lobectomy and radical lymph node dissection by single - port thoracoscope	1	Moderately	4.8×4.3
P03	R0	Thoracoscopic assisted right upper lobectomy and radical lymph node dissection	1	Unknown	4.3×5.1
P04	R0	Thoracoscopic assisted left upper lobectomy, pulmonary angioplasty, radical lymph node dissection	1	Moderately	2.7×2.3
P05	R0	Thoracoscopic assisted right upper lobectomy, bronchoplasty, radical lymph node dissection	1	Moderately	5.5×4.7
P06	R0	Thoracoscopic assisted right upper lobectomy and radical lymph node dissection	1	Unknown	3.7×2.1
P07	R0	Thoracoscopic assisted right middle and lower lobectomy and radical lymph node dissection	1	Unknown	3.8×3.3

### Molecular Genetic Features

**Figure 1:**
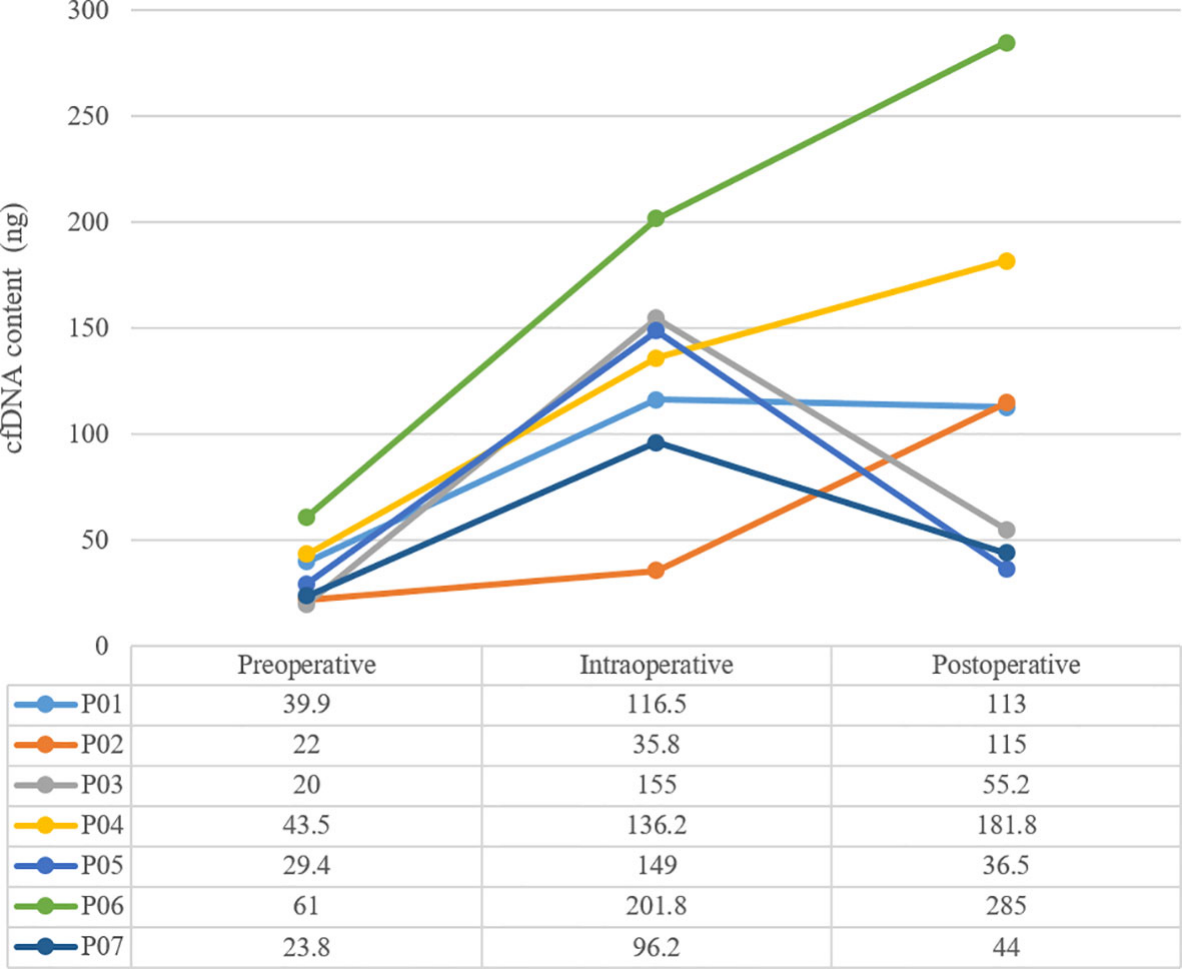
Perioperative dynamic monitoring of cfDNA content.

The mean sequencing depth of ctDNA detection is 7000-10000×, and 500-800× for tissue DNA detected. Mutations in tumor-related genes were detected in all 7 tissue samples. P01, P03, P06 and P07 were adenocarcinomas, and *EGFR* 19del or *EGFR* L858R mutation was detected. P02, P04 and P05 were squamous cell carcinoma, and *CDKN2A* and *TP53* were the main variants. Taking the tissue detection as the gold standard, the sensitivity and specificity of ctDNA detection in preoperative blood were 43.75% and 100%, respectively. The mutant allele frequencies (MAFs) in tissue detection were 32.38% ± 16.38%, and 1.50% ± 0.88% in preoperative blood. In one case (P03), the preoperative blood quality inspection was unqualified, so ctDNA state was not available. The test results are shown in **Table 3**. The total amount of cfDNA detection results are shown in **Figure 1**.

**Table 3:** NGS 23-gene panel detection results.

Patient No.	Gene	MAFs in tumor tissue	MAFs in peripheral blood		
			Preoperative	Intraoperative	Postoperative
P01	EGFR p.L858R	30.67%	0	0	0
	TP53p.S261Vfs*84	18.96%	0	0	0
P02	CDKN2A p.R58*	34.52%	1.33%	0	0
	TP53 p.R273L	29.98%	1.85%	0	0
P03	EGFR p.N771delinsK	47.78%	NA	0.98%	0.86%
	TP53 p.Y234C	75.33%	NA	0.34%	0.68%
P04	CDKN2A p.D74Rfs*44	42.40%	0.33%	0	0
	TP53 p.V272L	14.88%	0	0	0
	TP53 p.M237I	28.38%	0	0	0
P05	TP53 p.C229*	32.67%	1.61%	0	0
P06	EGFR p.L858R	18.67%	0	0	0
	EGFR p.E709V	19.93%	0	0	0
	TP53 p.M237I	4.18%	0	0	0
P07	EGFR p.L861Q	38.10%	2.50%	3.82%	0
	EGFR p.L858M	38.60%	2.44%	3.84%	0
	TP53 c.376­1G>T	43.10%	0.41%	1.75%	0

NA, not available.

### Survival Follow-Up Results

During the 18-month follow-up, P03, whose postoperative ctDNA was positive, and with decreasing cfDNA 7 days after surgical, had relapsed in December 2019 with multiple distant metastases and died of pleural effusion in July 2020. The disease-free survival (DFS) was 5 months and overall survival (OS) was 12 months. The remaining patients were followed up normally and no recurrence was found so far.

## Discussion

In recent years, precision medicine has been widely recognized in the field of internal medicine, as almost all medical diseases are related to heredity and genes. The treatment concept of classifying patients into different categories and administering different treatments according to molecular types based on genetic testing has significantly improved patients’ overall survival (OS). In the field of surgery, there are also numerous studies on the correlation between genotyping and prognosis prediction or recurrence risk assessment.

NSCLC is one of the malignant tumors with high morbidity and mortality in the world. With the wide application of targeted drugs such as *EGFR*-TKIs and *ALK*-TKIs, NSCLC has become the most developed tumor type of precision medicine field. Surgery is the most effective treatment for NSCLC, but the 5-year OS rate of stage I-IIIA NSCLC is about 38-74%, which means that 26-62% of postoperative NSCLC patients will relapse within 5 years (3).

To maximize the survival of patients with NSCLC, targeted drugs, chemotherapy and other adjuvant therapies are rationally used to deal with recurrence. But finding a solution to select patients with high risk of recurrence of close follow-up and timely intervention, as well as to identify patients with low risk of recurrence to avoid excessive treatment will be the developing direction of precision medicine in the surgical field.

ctDNA is a part of cfDNA derived from tumor tissues, the two share some characteristics: i) small amount, cfDNA in healthy people’s plasma is only about 18ng/ml, in NSCLC patients is up to 318ng/ml, and ctDNA may only account for 1% in cfDNA (8); ii) the fragments are very short, the lengths of them are usually only the sum of the length of the DNA wrapped around the nucleosome and the histone H1 joint, namely 166 base pairs; iii) with a short half-life about two hours, their status can be regarded as the real-time state of the tumor and the acquirement is relatively non-invasive, so they can be used for dynamic monitoring (9, 10).

Since ctDNA carries tumor genetic information, it has been used for precise therapy based on tumor molecular typing. However, the consistency between ctDNA and tissue DNA is still difficult to reach 100%. In this study, the MAFs of ctDNA were far lower than that of tumor tissue. Thus, even though the MAFs in the blood are low, there is still a high MAFs in the tissues. However, the MAFs of ctDNA in blood are more convincing considering that the MAFs of tumor tissue are greatly affected by spatial heterogeneity, and blood can be regarded as a sample of uniform texture. In this study, the specificity of ctDNA was 100%, which indicated that it could still guide the treatment correctly even with the low MAFs. Furthermore, the sensitivity of ctDNA was only 43.75%, which indicated that ctDNA testing might provide false negative results, and tissue testing should be considered to use as far as possible.

In the surgical field, ctDNA can be used to predict postoperative prognosis and assess the risk of recurrence. Previous studies found that patients with ctDNA detected immediately after radical resection in lung cancer, pancreatic cancer, breast cancer, colorectal cancer or other early stage tumors, had shorter DFS than those with undetected ctDNA, indicating that the prognosis of patients with detectable ctDNA after surgery was worse (11–14). The reason can be interpreted that the positivity of ctDNA indicates the presence of minimal (molecular) residual disease (MRD) that will increase the risk of recurrence. Other studies have confirmed that ctDNA with MAFs even < 0.1% can predict recurrence more than 100 days in advance of imaging in NSCLC patients, suggesting that ctDNA dynamic monitoring plays an important role in risk assessment of recurrence (15).

In one case (P07), the MAFs of ctDNA were 1.78% ± 1.19% before surgery and increased to 3.14% ± 1.20% in intraoperative blood, but ctDNA was undetected in postoperative blood. This phenomenon may be explained by a large amount of ctDNA was released into the blood under the influence of surgery, that resulted in a transient increase in the MAFs of ctDNA. The different types of surgical operation, the degree of pressure loaded onto the tumor, and the disconnection sequence of arterial and vein may affect the transient increase of ctDNA. Evidence for whether this abundance increases has an effect on the postoperative ctDNA level and whether it has a predictive effect on the recurrence after surgery is still lacking.

In addition, only one (14.3%) patient (P03) was tested positive about ctDNA in 7 days after surgery. But considering the reported 5-year recurrence rate of NSCLC was about 26-62%, it indicated that using ctDNA alone as the predictor may miss some “high-risk” patients. The low positive rate of ctDNA might be the false negative results caused by the extremely low MAFs of ctDNA. As we know, the amount of ctDNA at each TNM stage of NSCLC is different, with lower level in early stage and higher level in later stage. For some early NSCLC patients, the levels of ctDNA are even too low to be detected by NGS after surgery. In that case, false negative will forms. On the other hand, the key to distinguish ctDNA from cfDNA depends on detection of tumor-related somatic variation, so the positive rate of ctDNA is closely related to the size of targeted area in genotyping and the sensitivity of the detection method. In terms of NGS, the panel size used for detection and the sequencing depth are two crucial factors of positive rate of ctDNA.

Be there any better markers that can be used as an independent predictor of recurrence risk? Or can it was combined with ctDNA together to predict recurrence risk? It was reported on one previous study in 2001, the concentration of cfDNA in NSCLC patients was much higher than that of healthy people before surgery, and returned to the normal level in 6 months after surgery, then increased again when recurrence occurred, which suggested that concentration of cfDNA also has the potential to dynamically monitor recurrence (8). In this study, we did not use healthy people’s cfDNA as control, but we observed that the amount of cfDNA immediately increased after the operation from preoperative level of 36.6 ± 14.7ng to 127.2 ± 52.2ng. However, this phenomenon showed polarization in 7 days after the operation: the amount of cfDNA from P03, P05 and P07 showed a significant decline trend, and basically returned to the preoperative level. Meanwhile, the amount of cfDNA from P01, P02, P04 and P06 remained at a high level or even continued to rise. Since 10ml of peripheral blood was extracted exactly from each patient, the total amount of cfDNA extracted each time could be regarded as an approximate indicator of cfDNA concentration.

cfDNA is typically about 166 bps of double-stranded DNA, mainly released from cell damage, including cell lysis, necrosis and apoptosis. Under the stress of surgery, the level of cfDNA will increase, but with the completion of surgery and the healing of tissue wound, cfDNA should be restored to the preoperative level like P03, P05 and P07. If the operation causes more trauma to the tissue, the wound healing will be slower, and the stress will lasts longer, it may indicate an increased risk of tumor hematogenous dissemination or recurrence, and the early pattern of the manifestation is likely to be the sustained high amount of cfDNA in patients such as P01.

Follow-up data onto the later period of this study also showed that only one patients (P03) with positive ctDNA after surgery and decreasing cfDNA, had relapsed quickly after surgery, DFS was only 5 months and OS was 12 months. This phenomenon presented us a possibility: the ctDNA could be detected even after the reduction of cfDNA after surgery, suggesting that the absolute amount of ctDNA was probably large, and those patients also had the highest risk of recurrence, such as P03 in this study. On the contrary, the amount of cfDNA was elevated to some patients after surgery, but ctDNA could not be detected, such as P02, P04 and P06. Did it indicate that the tumor was removed completely from MRD residue, and the risk of recurrence is reduced?

In summary, we observed the dynamic changes of ctDNA and cfDNA in early NSCLC during perioperative period with a small sample size in this study, and discussed the consistency between blood gene detection and tissue gene detection. The bipolarization of cfDNA concentration on perioperative periods has been found for the first time, and dynamic changes of cfDNA combined with ctDNA may be a new predictive biomarker for recurrence in early NSCLC. However, due to the limited sample size and relatively short follow-up time, its predictive efficacy still needs to be verified.

## Data Availability Statement

The data used to support the findings of this study have been deposited in the NCBI repository (https://www.ncbi.nlm.nih.gov/sra/?term=PRJNA740302).

## Ethics Statement

The studies involving human participants were reviewed and approved by the ethics committee of Daping Hospital affiliated to Army Medical University. The patients/participants provided their written informed consent to participate in this study.

## Author Contributions

XZ and DH participated in study design, data analysis and article writing. FD and LM played roles in the surgical treatment and collection of follow-up data of patients. XShen, HZ, and XShe participated in data analysis and article writing. ZM played a role in surgical guidance and overall planning. All authors contributed to the article and approved the submitted version.

## Conflict of Interest

Author DH, XS, HZ and XS were employed by the company 3D Medicines Co., Ltd.

The remaining authors declare that the research was conducted in the absence of any commercial or financial relationships that could be construed as a potential conflict of interest.
